# Three potential challenges in studying COVID-19 pandemic data: Chinese statistics, social media, and preprint servers 

**Published:** 2020

**Authors:** Mohamad Amin Pourhoseingholi, Sajad Shojaee, Sara Ashtari

**Affiliations:** 1 *Gastroenterology and Liver Diseases Research Center, Research Institute for Gastroenterology and Liver Diseases, Shahid Beheshti University of Medical Sciences, Tehran, Iran *; 2 *Basic and Molecular Epidemiology of Gastrointestinal Disorders Research Center, Research Institute for Gastroenterology and Liver Diseases, Shahid Beheshti University of Medical Sciences, Tehran, Iran*

 Coronavirus (COVID-19), which started in Wuhan, China, in early December 2019, has now become the biggest challenge to health for almost all countries in the world. On January 31, 2020, the WHO declared COVID-19 a public health emergency, and then on March 11, COVID-19 was officially declared a pandemic. To overcome this worldwide outbreak, scientists and health policy makers around the world have tried to implement prevention strategies (e.g., home isolation, travel restrictions, social distancing, etc.) or have begun research projects for the development of a vaccine and new drugs against this novel coronavirus. Many studies are already focusing on the epidemic, the mechanism of the novel coronavirus, the medication, the epidemiology of infection, etc., and most scientific journals have begun fast-tracking manuscripts regarding COVID-19; many papers have been published since the epidemic began. However, there are some challenges (despite financial resources or the complexity and unknown nature of the virus) which could potentially lead to misleading and incorrect results and decisions. The first challenge is the Chinese statistics; the first report of COVID-19, the statistics of infected persons and mortality, fatality rates, and then the first published manuscripts all came from China and is being used as the basic information on the epidemiology of the novel coronavirus. Now, however, new criticism has been raised against the Chinese reports. The Chinese information (specifically, numbers of infected and death cases) is clouded by a fog of skewed data, political imperatives, and unreported cases and possibly deaths. The underestimation of Chinese statistics is not just a claim; at the start of the epidemic in Wuhan, it was predicted that the most plausible number of infected cases was in the thousands (rather than the hundreds reported by Chinese authorities), and even this is likely an underestimate (1). Therefore, this skeptical data put other nations at risk in adequately understanding and preparing for the disease and caused researchers and policymakers to underestimate the fatality and severity of the epidemic. 

The second and third challenges for COVID-19 studies go hand in hand with each other: the preprinted manuscripts regarding coronavirus and social media (or even official media) which provides the general population with information and breaking news about the pandemic. In this particular outbreak, authors were encouraged to share their manuscripts on preprint servers before the process of peer review. Thus, any findings regarding COVID-19 were immediately posted online without external checks or validation. While making COVID-19 studies visible is highly useful (if the data is accurate), false or fraud data could be much worse not only for researchers who use the data (which did not pass the peer-review process), but also for people in the general population. Some of these preprint manuscripts have been shared widely on social media and picked up by official media as headlines, which further spread the findings to the public. To date, a total of 3077 articles (2476 on medRxiv server and 601 on bioRxiv server) have been uploaded as preprint manuscripts in the domain of COVID-19 SARS-CoV-2 (2). Some of these manuscripts have been cited while they have not yet been published. Moreover, the spread of unconfirmed information or misleading science by (social) media can create panic and may make a disease epidemic worse by prompting false policy actions or encouraging people in risky behaviors. In this situation, the role of the (social) media is more prominent. In the pandemic outbreak of COVID-19, social media has been a double-edged sword. There are billions of smart phones and footprints from web searches and digital data (which, however, still remains largely inaccessible to researchers and governments). Positively, the data could support community surveillance, contact tracing, social mobilization, and health promotion during the COVID-19 pandemic (3). If responsibly and appropriately used, social media can provide rapid and effective dissemination routes for key information (4). Negatively, however the “Infodemic” (as it is termed by the WHO) or fraud information can damage the right decisions, behaviors, and actions. The pandemic of social media panic may travel faster than the COVID-19 outbreak (5). Its casualty would increase even more than the burden of COVID-19. The outbreak of methanol mass poisoning is a bold example of how false information (the effectiveness of drinking alcohol against COVID-19) spread in social media (Iran) can lead to substantial mortality and morbidity (in some provinces of Iran, the rates were higher than that of COVID-19) (6). 

In conclusion, stakeholders' participation is required to reduce the impact of fake news during this pandemic (7). Public health institutes could combat misinformation by updating key facts, using their official media platforms (8); health professionals should cooperate with the mass media to help reduce the fraud information (9); and last but not the least, researchers themselves should pay more attention to the data and information extracted from the mass waves of COVID-19 manuscripts from preprint data or from skeptical Chinese statistics. 
